# Bias at the Bedside: A Comprehensive Review of Racial, Sexual, and Gender Minority Experiences and Provider Attitudes in Healthcare

**DOI:** 10.3390/healthcare14010114

**Published:** 2026-01-03

**Authors:** Emily J. R. Carter, Roberto Sagaribay, Aditi Singh, Lorraine S. Evangelista, Deborah A. Kuhls, Jennifer R. Pharr, Kavita Batra

**Affiliations:** 1 Office of Research, Kirk Kerkorian School of Medicine at UNLV, University of Nevada, Las Vegas, NV 89102, USA; emily.carter@unlv.edu; 2 School of Public Health, University of Nevada, Las Vegas, NV 89119, USA; 3 Department of Internal Medicine, Kirk Kerkorian School of Medicine at UNLV, University of Nevada, Las Vegas, NV 89102, USA; aditi.singh@unlv.edu; 4 Sue & Bill Gross School of Nursing, University of California, Irvine, CA 92697, USA; l.evangelista@uci.edu; 5 Department of Surgery, Kirk Kerkorian School of Medicine at UNLV, University of Nevada, Las Vegas, NV 89102, USA; deborah.kuhls@unlv.edu; 6 Department of Environmental and Global Health, School of Public Health, University of Nevada, Las Vegas, NV 89119, USA; jennifer.pharr@unlv.edu; 7 Department of Medical Education, Kirk Kerkorian School of Medicine at UNLV, University of Nevada, Las Vegas, NV 89102, USA

**Keywords:** healthcare disparities, discrimination, implicit and explicit bias, minority stress, structural racism, intersectionality, sexual and gender minority health, patient-provider communication

## Abstract

Background/Objectives: Persistent inequities in healthcare experiences and outcomes among marginalized racial/ethnic groups and sexual and gender minority (SGM) populations have been well documented. However, disparities in perceptions of discrimination and bias between patients and health providers remain insufficiently understood. This review synthesizes the current evidence on how these groups differently perceive discrimination, how bias manifests in clinical encounters, and how intersecting identities shape healthcare experiences. Methods: A comprehensive review using SANRA guidelines examined racial/ethnic discrimination, SGM-related bias, provider implicit attitudes, minority stress processes, and structural determinants of inequity in healthcare settings. Articles were identified through systematic search strategies across major databases, and their conceptual, methodological, and theoretical contributions were analyzed. Results: Across studies, marginalized patients consistently reported discrimination, stigma, and mistrust in healthcare, whereas providers often underestimated the prevalence and impact of these experiences. Evidence indicates that implicit pro-White biases among providers influence communication quality, clinical decision-making, and patient comfort. Structural racism and intersecting minority statuses further compound disparities, contributing to delayed care, unmet health needs, and poorer outcomes. Limited alignment between patient and provider perceptions suggests a gap in recognition of inequitable treatment and its drivers. Conclusions: Healthcare inequities arise from interconnected, interpersonal, and structural mechanisms. Addressing these disparities requires multilevel interventions targeting provider training, institutional policy reform, and system-level barriers. Integrating both patient and provider perspectives is essential for developing equitable, affirming models of care and improving health outcomes for racial/ethnic and SGM populations.

## 1. Introduction

Healthcare inequities are a persistent public health issue globally [1,2]. Marginalized populations, including racial and ethnic minorities as well as Lesbian, Gay, Bisexual, Transgender, and Queer or Questioning (LGBTQ+) communities, are disproportionately impacted. These populations report increased levels of stigma and discrimination with barriers to healthcare compared to individuals in the general population [1,3]. These inequities are associated with higher morbidity and mortality [2,4], as well as adverse mental health outcomes. National surveys in the United States indicate that nearly 40% of LGBTQ+ racial minority individuals report experiencing discrimination when seeking healthcare, significantly higher than the rates reported by White, heterosexual individuals [1,5,6,7]. On a global scale, these marginalized individuals have an increased vulnerability to chronic illnesses, disease complications, and mental health disorders [6,8,9], which contribute to systemic barriers to healthcare access [6,10].

**Figure 1:**
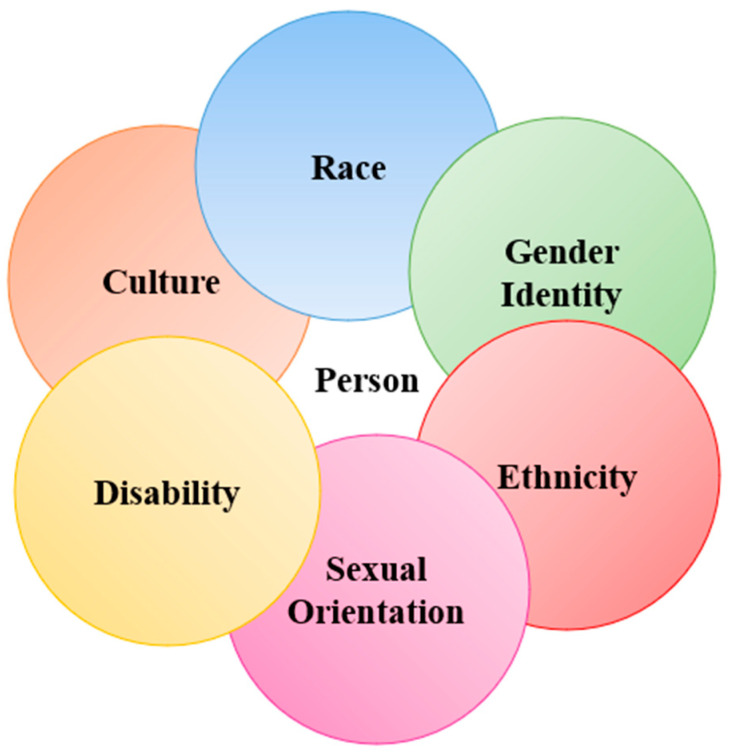
Diagram of intersecting identities. Visual representation of the multiple identities that contribute to a person’s unique experiences. This illustration is a non-comprehensive example; this diagram emphasizes the major identities discussed in this review.

The inequities are rooted in enduring stigma and structural racism. The literature expresses the persistence of inequities through many examples. For instance, Black and Hispanic patients have reported higher mistrust in medical services and decreased satisfaction with care compared to White individuals [2,4,11]. LGBTQ+ adults report delaying their healthcare due to fears of stigma and discrimination [12,13]. Transgender individuals report higher levels of unmet healthcare needs [12,14,15]. These axes at which marginalized identities intersect (Figure 1) can exacerbate the impacts of disparities and structural inequities [16,17,18].

This compounding effect is evident in how implicit and explicit biases undermine patient-provider relationships and can perpetuate mistrust [19,20,21,22]. Systematic reviews on this topic have consistently shown that healthcare providers display pro-White implicit biases when compared to the general population [23,24,25]. This is associated with poorer communication, poorer outcomes, and reduced adherence to treatment for racial and ethnic minority patients [23,24,26]. However, these inequities are found beyond racial discrimination. Sexual and gender minority (SGM) patients experience denial of care, misgendering, and heteronormative assumptions, which in turn compromise access to affirming services [1,27]. These challenges contribute to SGM patients avoiding care, concealing identity, and increased mental health burdens [12,28,29].

While the body of research on this topic is expanding, notable gaps remain to be addressed. Studies often only examine either the patient experience or the provider experience in isolation. These gaps limit opportunities to fully understand how bias is displayed and perceived across both the patient and provider ends of a patient encounter [8,30]. The literature has shown evidence that discrimination fosters minority stress and delays care [3,4,5]. The provider focus research shows how attitudes and implicit biases, shaped by many factors including training, cultural competence, and religiosity among others, continue to influence decision-making and clinical communication [24,31,32,33]. Both perspectives, together, provider and patient, provide a more holistic understanding of how interpersonal and systemic inequities develop healthcare disparities [19,25].

This review pursues two aims. The first aim is to synthesize literature on the lived experiences of healthcare users from racial/ethnic and SGM groups with attention to discrimination, patient reactions, and overall health impacts. The second aim is to explore the biases, attitudes, and perceptions of healthcare providers, including the variability in actions and responses and structural inequities within the workforce. These aims together elucidate recognition of inequities and differences in patients’ mistrust of the healthcare system, with providers’ focus on personal behaviors [20,23].

This review seeks to apply and understand the Minority Stress Theory, Structural Racism frameworks, and Intersectionality to document inequities and contextualize them within the broader context of societal discrimination and stigma [17,34,35]. Applying Bernard Weiner’s Attribution Theory also offers insight into how individuals interpret and respond to inequitable attributions. The attribution theory explains that people assign causes to events, internal or external and stable or unstable, in turn shaping their emotions and overall behaviors [36,37]. Among healthcare settings, the attributions influence how both patients and providers explain bias [36,38].

The frameworks applied in this review are central to understanding the structural underpinnings and psychological inequities discussed. The Minority Stress Theory [5] clarifies how chronic exposure to discrimination and stigma leads to coping strategies and stress-related health outcomes, as a recurring pattern among sexual and gender minority patients. For example, repeated exposure to discrimination may lead SGM patients to avoid preventative care, increasing risk for unmanaged chronic illness or delayed cancer diagnosis. The Structural Racism Theory [2] explains how interpersonal biases in institutional systems can perpetuate inequity through training, resource distribution, and policy. For instance, institutional policies and resource allocation practices can systematically disadvantage racial minority patients through reduced access to specialty care or longer wait times. Furthermore, Intersectionality [16] explains how overlapping identities, including sexuality, race, and gender, can create compounded vulnerability, and can be used to help interpret studies where patients’ multiple identities amplify stigma. As an example, a Black transgender patient may experience compounded barriers arising simultaneously from racism, transphobia, and gendered assumptions in clinical encounters. Lastly, the Attribution Theory [36] can be applied to understand how providers and patients cognitively interpret the cause of discrimination differently, and how this can impact communication and trust. The theories together can guide the synthesis and interpretation of findings in this review.

This analysis is anchored in important concepts aimed at pursuing equity, workforce training, and systemic change by linking patient and provider perspectives [39]. Despite extensive literature documenting healthcare discrimination, few reviews integrate both patient and provider perspectives within shared theoretical frameworks, limiting understanding of how perceptual gaps contribute to persistent inequities. The goals of this review are to inform future interventions that promote equity, patient-provider relationship trust, and advance healthcare delivery to racial and SGM populations.

## 2. Methods

This comprehensive review was guided by two primary research questions to understand both dimensions of racial sex and gender minority discrimination and stigma in healthcare.

- What are the experiences of “persistent racism”, defined here as repeated and enduring experiences of discrimination embedded within healthcare interactions and institutional practices over time, among healthcare users in sexual minority groups in medical settings?
  - What are the associations between perceived racism in healthcare settings and perception of illness among healthcare users at the intersections of race, gender identity, and sexual orientation?
- What are the attitudes and perceptions of healthcare providers towards racial interaction or climate in healthcare?

### 2.1. Literature Search

This comprehensive review was conducted from 28 August through 16 September 2025 in accordance with the SANRA (Scale for the Assessment of Narrative Review Articles) guidelines [40]. A comprehensive approach was used to synthesize the data and analyses that comprise the perceptions of both healthcare users and providers among patient–provider interactions in clinical settings for individuals of racial and sex-and-gender minorities. To capture the breadth of literature, PubMed, Embase, and Google Scholar were searched without year limits and restricted to English-language-only studies. A separate literature search was conducted for healthcare users and healthcare providers with different search terms relevant to the population. The following search terms were used for the healthcare users literature search: (racism OR healthcare disparities OR discrimination OR implicit bias) AND (“sexual and gender minorities” OR LGBTQ) AND (health services OR healthcare OR delivery of healthcare) AND (patients OR patient perspective OR healthcare user). The following search terms were used for the healthcare providers literature search: (healthcare providers OR physicians OR nurses OR clinicians OR medical trainees OR health professionals) AND (attitudes OR perceptions OR beliefs OR bias OR “implicit bias” OR “explicit bias” OR “cultural competence” OR “provider-patient relations” OR “workplace climate”) AND (racism OR “racial discrimination” OR “racial bias” OR “structural racism” OR “racial prejudice” OR “race relations”) AND (quantitative OR survey OR questionnaire OR “cross-sectional” OR “psychometric” OR “scale” OR “instrument”) AND (“sex and gender minority” AND LGBTQ AND gay AND lesbian). The reference lists of key papers were cross-analyzed to identify additional papers. The healthcare user literature search before screening yielded 2854 studies before screening. The healthcare provider literature search before screening yielded 1546 studies before screening.

### 2.2. Inclusion and Exclusion Criteria

Studies that were included are those that (a) were published in peer-reviewed journals, (b) included analyses focusing specifically on racial or sex and gender minority patients experiences interacting with healthcare, (c) included analyses focusing specifically on healthcare providers experiences, behaviors, and attitudes towards interacting with racial or sex and gender minority patients, (d) included analyses of healthcare provider’s perceptions of racial interaction within the clinical setting, (e) singular identity and multiple, intersecting identity analyses. Sources excluded include those that did not directly analyze either healthcare users’ or healthcare providers’ perspectives on racial or sex- and gender-minority experiences in healthcare. Additionally, sources that did not investigate the experiences and perceptions among racial, sex, and gender minority patients, and focused on patients without specifying these identity characteristics. Sex and gender minority patients include those individuals whose sexual orientation, gender identity, gender expression, or sex assigned at birth differ from societal norms tied to cisgender and heterosexual identities. This includes lesbian, gay, bisexual, transgender, nonbinary, queer, and other diverse identities that experience marginalization and health inequities. Screening and selection were done by one reviewer. 64 articles met inclusion criteria and were included in this review. Additional articles that were found upon search later in writing the manuscript were also included in this review.

### 2.3. Study Scope

The reviewed literature on healthcare inequities among racial/ethnic and sexual and gender minority populations includes a variety of methodological approaches, including several cross-sectional surveys capturing the prevalence of stigma, discrimination, and implicit bias in healthcare encounters [1,22,23,24,25]. The surveys together provide broad insights into patterns of inequities in a variety of regions and clinical settings. This highlights disparities in communication quality, health-avoidance behaviors, and denial of care [4,27,41]. However, findings are also shaped by variations in survey design, context-specific measures, definitions of bias, and complicated comparability across studies [19,20,24].

In addition to survey studies, there are qualitative and mixed-methods studies. Qualitative interviews, including focus groups with LGBTQ+ and minority patients, have revealed common narratives of concealment, avoidance, misgendering, and self-advocacy, showing how stigma is internalized and negotiated in everyday healthcare experiences [28,42,43]. Among the literature are some mixed-methods studies that combine surveys with open-ended responses, helpful in identifying intersectional discrimination and compounding effects of gender identity, race, and illness status [44,45,46]. These studies are less generalizable due to smaller samples; however, they provide depth in the mistrust, emotional burdens, and coping strategies that quantitative surveys do not fully capture.

Regarding literature on healthcare providers’ perceptions and attitudes, experimental and simulation-based studies have proven useful tools for examining implicit bias in real time. Several studies have used the Implicit Association Test (IAT). Other studies have used the clinical vignette-based decision-making scenarios and standardized patient encounters to highlight unconscious bias manifesting in physical language, nonverbal synchrony, and empathy [23,38,47,48,49]. The methods described have enabled researchers to influence provider attitudes in controlled conditions, yet they do not fully reflect the complexity of real-world interactions.

Furthermore, systematic and scoping reviews have provided important syntheses of the patterns of bias and healthcare outcomes in this research topic. Reviews show patterns of implicit pro-White bias among healthcare providers, disparities in patient-provider relationships through communication, and poor health outcomes among vulnerable minority populations [8,9,19,20,24]. The reviews have underscored the ways that stigma contributes to delayed care, worsens survivorship outcomes in cancer, and has a negative impact on mental health among patients [12,43,50]. Therefore, this review highlights the literature and research done to document inequities as well as the gaps that remain in understanding structural racism and intersectionality in healthcare delivery [2,4].

Altogether, the literature demonstrates that methodological approaches contribute to the depth and strength of research findings: surveys provide breadth, qualitative research provides depth, and simulation methods provide a way to view mechanisms of bias in practice. The diversity of methodological approaches underscores the need to triangulate the evidence to capture the lived experiences of healthcare users and the underlying behaviors and attitudes of healthcare providers. The multi-method body of literature contributes to the comprehensive understanding of the need to address inequities in healthcare at the interpersonal and systemic levels.

### 2.4. Analytical Approach

**Figure 2:**
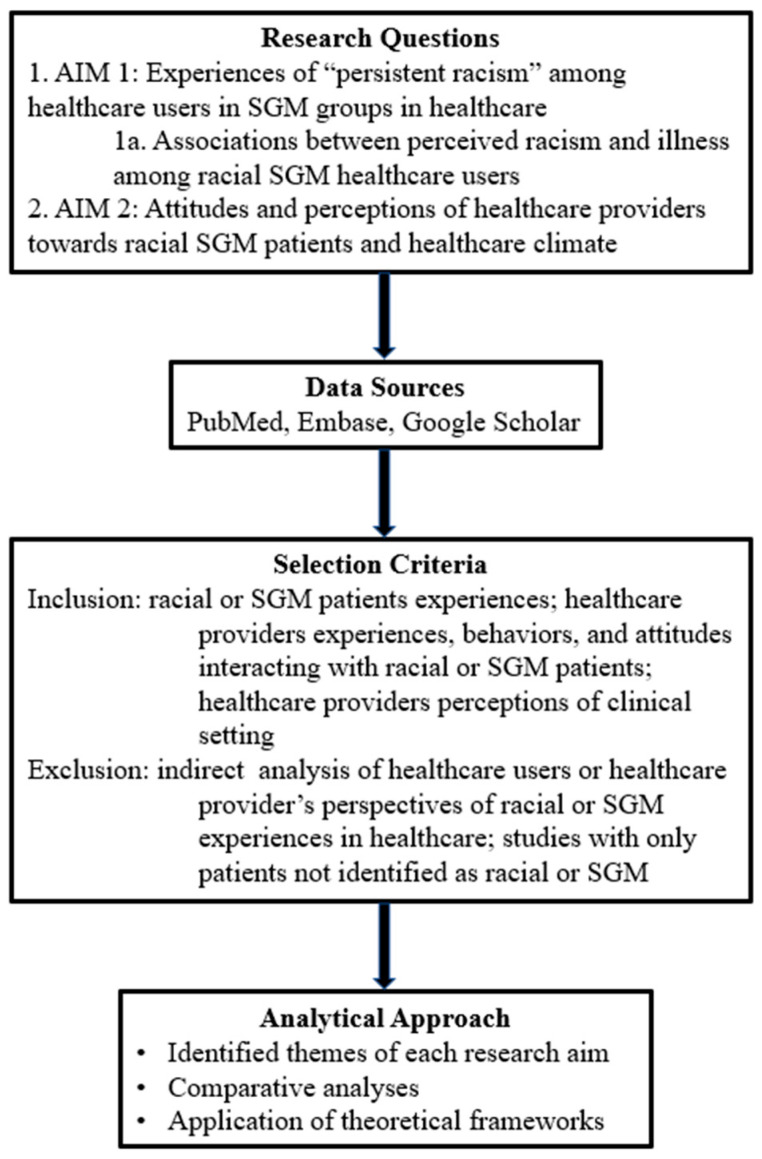
Conceptual framework of the comprehensive review. This diagram illustrates the structure and flow of this extensive review, including research questions, data sources, selection criteria, and analytical approach. Includes the variety of data sources used to identify studies, a comprehensive overview of the requirements for the review, and a summary of the approach taken to investigate the research questions.

The findings of this comprehensive review are thematically synthesized based on the two aims (Figure 2). The themes identified for this review include those repeated and identified throughout the literature included in this analysis. For the first aim, the themes are based on patient-centered literature on marginalized populations, which encompass: (1) experiences of discrimination and stigma, (2) coping mechanisms and adaptive strategies, (3) health impacts of bias and avoidance, (4) communication gaps in clinical encounters, and (5) intersectional dimensions of race, gender, illness status, and sexuality. For the second aim, the themes are derived from provider-focused studies, which encompass: (1) implicit and explicit biases, (2) knowledge and training gaps, (3) attitudinal variability influenced by prior exposure, geography, and religion, (4) communication behaviors and subtle manifestations of bias, and (5) workplace dynamics and structural inequities.

In these domains, the results are from across study designs, including quantitative surveys, qualitative interviews, mixed-methods research, simulation studies, and systematic reviews, across many intersectional identity populations. A comparative approach highlights both convergences, including reports of discrimination from patients and acknowledgement of implicit bias among providers. However, there are many divergences, such as providers underestimating the frequency and impact of stigma compared to patient reports.

This synthesis also produced methodological strengths and limitations. The surveys provide breadth on documented inequities, and qualitative studies offer insights into patients’ lived experiences and coping mechanisms. Simulation-based research identified mechanisms of bias in controlled environments. Triangulating this evidence across all these methods, this review can capture not only what is known about patient and provider perceptions of healthcare inequities, but also the research practices shaping the findings. During the synthesis process, applying theoretical frameworks can help organize findings and lenses (Table 1). The Minority Stress Theory can provide a framework for analyzing patients’ coping and avoidance behaviors. At the same time, the Structural Racism Theory can inform an examination of institutional barriers and policy-level inequities. An intersectional interpretation of the findings can identify where multiple marginalized identities collaborate, and the Attribution Theory can provide a more informed comparative interpretation of provider and patient narratives to explain divergent perceptions of blame, empathy, and responsibility. SANRA was used as a guiding framework while developing this review to ensure the importance of the topic is clearly justified, there are clear aims, literature search is thorough, and there is scientific reasoning with an evidence-based approach.

Although this review incorporates a wide range of study designs, the strength of evidence is constrained by methodological limitations across the included literature. Much of the existing research relies on studies that are cross-sectional surveys, which are useful for capturing prevalence estimates of discrimination and bias, but cannot establish causality or temporal ordering of events. Many of the included qualitative studies are drawn from small, convenience samples, often from single geographic regions or institutions that limit generalizability. Many of the provider-focused studies employ simulation-based designs that include standardized patient encounters and vignette experiments, which offer valuable insights into mechanisms of bias but do not fully capture the complexity of real-world clinical interactions. Furthermore, measurement tools, such as the Implicit Association Tests (IATs) and self-reported discrimination or stigma scales, vary widely in the operational definitions and may introduce social desirability or response bias. All together, these factors suggest the strength of evidence is moderate and limited in its ability to infer causality, system-level effects, and long-term outcomes, underscoring the need for more multi-site, longitudinal, and intersectional grounded research.

## 3. Results and Discussion

### 3.1. Perceptions of Healthcare Users

#### 3.1.1. Experiences of Discrimination and Stigma

SGM patients, especially those of a racial/ethnic minority, continue to report in the literature lived experiences of discrimination in healthcare. Such experiences include misgendering, denial of care, and biased treatment from clinical and non-clinical staff [1,13,29,44,51]. For example, a study in Turkey reports that nearly half of LGBTQ+ youth reported experiencing identity-based discrimination [51]. Transgender patients experienced the highest levels of mistreatment and refusal of gender-affirming care [51]. Similarly, evidence from a systematic review indicated that discrimination and stigma contribute to service refusal, verbal and physical abuse, and disproportionate discrimination and burden among transgender patients [1]. Furthermore, in a study on palliative and supportive care services among LGBTQ+ seriously ill patients and partners, participants reported disrespectful and inadequate care [44]. These experiences, among many others, illustrate the interpersonal and systemic stigma that create unsafe environments for SGM patients. These findings reflect the processes outlined in the Minority Stress Theory [5] through which chronic exposure to invalidation and discrimination produces cumulative psychological stress, further reinforcing avoidance of healthcare. These experiences, echoed in many studies, exemplify the social determinants framework embedded within the Structural Racism Theory [2], demonstrating how inequities are sustained by institutional and cultural norms, not isolated prejudice.

#### 3.1.2. Coping Mechanisms

Patients often develop coping behaviors to mitigate the effects of discrimination, yet these strategies may inadvertently exacerbate inequities in care over time. A study of LGBTQ+ and Black, Indigenous, and People of Color (BIPOC) patients observed strategies that patients adopted, such as concealment of identity, avoidance of certain providers and healthcare services, and excusing discriminatory behaviors by attributing the behaviors to stress and overwork [28,42,52]. Participants also reported self-advocacy, where patients would challenge providers directly due to discrimination [28]. It is important to understand that, while the coping mechanisms developed and enacted upon provide temporary relief and exit from challenging situations, this reinforces cycles of mistrust and discrimination and eventual withdrawal from seeking healthcare [5,29]. Patients in challenging healthcare settings, including cancer care, described the need to actively self-advocate and balance disclosure of their identity with fear of mistreatment, leading to large emotional tolls in navigating clinical encounters [42]. In the context of the Minority Stress Theory [5], coping behaviors illustrate maladaptive and adaptive stress responses to stigma. Concealment and avoidance can provide short-term protection; however, they also perpetuate disconnection from healthcare systems and support the theoretical notion that minority stress contributes to long-term health inequities.

#### 3.1.3. Health Impacts

Unfortunately, the consequences of discrimination go beyond negative encounters and surface-level outcomes to impact health status. Many studies report delayed or avoided healthcare as a common response among LGBTQ+ populations, driven by stigma and fear of being misgendered [41,53,54,55]. Transgender patients experience greater levels of healthcare avoidance and internalized stigma, which leads to worse mental health outcomes and less adherence to treatment plans [12,56]. A systematic review of SGM cancer care identified higher levels of distress, decreased survivorship outcomes, and inadequate support for LGBTQ+ patients compared to heterosexual patients [43]. These findings underscore how the overall stigma in healthcare settings perpetuates inequities in mortality and morbidity among SGM patients.

#### 3.1.4. Communication Gaps

Despite providers’ efforts to consciously reject stigma and prejudice, implicit bias manifests through language, empathy, and other nonverbal behaviors [30,38,47,48,49]. Physicians with greater implicit bias scores were more likely to use anxiety-related words or pronouns, showing dominance during racially discordant interactions, contributing to a less positive patient experience [30]. Simulated encounters also demonstrated that physicians with pro-White implicit bias tended to communicate more effectively with White standardized patients than with Black patients, most often in rapport building and information gathering [47]. Studies also show that nonverbal behaviors reflect bias. For instance, in oncology settings, implicit bias has been linked to reduced patient-provider trust and altered nonverbal synchrony, shaping the subtle dynamics of clinical encounters [48]. These findings demonstrate that bias operates at the micro-level of communication and may reinforce disparities even without overt prejudice.

#### 3.1.5. Intersectionality

The intersectionality of sexual orientation, gender identity, race/ethnicity, disability, and illness compound the many barriers encountered by SGM patients [16,17,44,45,47]. The intersectional analyses of these axes demonstrate that patients with multiple marginalized identities face amplified risks of overall stigma and discrimination. For instance, studies of LGBTQ+ persons who are also BIPOC have documented disproportionate exposure to power inequities and biased medical decision-making embedded within systemic discrimination [45]. Black and Latino patients also report covert and overt racialized bias in clinical encounters, emphasizing mistrust and avoidance of healthcare, particularly shaped by socioeconomic status and race [57]. LGBTQ+ patients who are seriously ill experience intersectional discrimination that is compounded by illness stress [44]. This underscores how the multiple axes of marginalization produced layered vulnerabilities in healthcare settings for patients. The application of Intersectionality [16] strengthens the interpretation here by acknowledging that gender, race, and sexuality interact dynamically rather than independently. Through this perspective, it can be explained why the magnitude and nature of discrimination differ for patients on multiple social margins, revealing the compounded effects of interpersonal and structural stigma.

### 3.2. Perceptions of Healthcare Providers

#### 3.2.1. Implicit and Explicit Bias

A large body of literature demonstrates that healthcare providers, comparable to the general population, have implicit biases that favor White, heterosexual, and cisgender patients [19,20]. Systematic reviews further confirm that implicit racial bias is prevalent among healthcare professionals and trainees [19,47]. This is overall associated with poorer patient-provider communication and worse patient outcomes [19,20,22,23,25]. Numerous Implicit Association Test (IAT) studies have demonstrated that healthcare providers exhibit significant pro-White implicit bias, frequently exceeding levels observed in the general population [32,58,59]. Although explicit bias is less prevalent, it is not absent. Surveys of physicians in Canada revealed explicit and unfavorable attitudes towards Indigenous patients [60,61]. Similarly, dental hygienists in the United States demonstrate moderate levels of color-blind racial attitudes with a lack of awareness of structural racism’s effects on patient care [62,63]. Collectively, these findings reveal that implicit and explicit biases, whether recognized or unacknowledged, profoundly influence the quality and equity of clinical interaction. The findings align with the Structural Racism Theory [2], which holds that bias among providers does not exist in isolation and is reinforced by institutional norms, organizational hierarchies, and medical curricula. Through the Attribution Theory [36], further implicit tendencies can also be understood as cognitive shortcuts in shaping clinical judgement, in which providers may unconsciously attribute patient outcomes to individual behavior rather than systemic inequity.

#### 3.2.2. Knowledge & Training Gaps

Deficits in training and knowledge about LGBTQ+ and minority health remain a significant barrier to creating equitable healthcare [31,33,64,65]. A mixed-method systematic review of mental health practitioners found that most providers had positive attitudes towards LGBTQ+ patients and reflected inadequate knowledge with discomfort in discussing gender identity with their patients and lack of confidence in delivering affirming care [31]. A study from Greece reported that over 80% of healthcare professionals expressed willingness to receive training on LGBTQ+ health issues and terminology to promote equitable care [33]. Medical education globally has curricula that often emphasize cultural competence in a narrow sense, and insufficient focus on structural racism, intersectionality, and SGM-specific health needs [64]. Addressing these persistent educational gaps is essential to advancing health equity, ensuring that all providers possess the competence and confidence to deliver inclusive, affirming, and evidence-based care. The persistence of these knowledge deficits underscores Structural Competency [63], an extension of the Structural Racism Theory [2]. These findings emphasize the need for provider education that links patient outcomes to systemic structures rather than cultural differences alone.

#### 3.2.3. Attitudinal Variability

Multiple factors, including prior exposure, sociocultural context, religiosity, and gender, shape providers’ attitudes toward minority patients [31,32,33,59,66]. Evidence suggests that providers with greater religiosity or conservative political orientation are more likely to express bias and discomfort towards LGBTQ+ patients. In contrast, providers with personal or professional connections to LGBTQ+ individuals have more affirming attitudes [31,33]. Studies have also shown geographic variation in implicit biases among nurses and physicians. Bias scores were highest among nurses in the Midwest and physicians in the Southern U.S., reflecting regional sociocultural influences [24,32,67]. Therefore, provider perceptions cannot be generalized uniformly across professions or regions. Interventions have to be tailored to account for these variabilities in attitudes [25,68]. Regional and sociocultural variation can also be conceptualized through the Attribution Theory [36], which posits that providers embedded in certain sociopolitical contexts may internalize explanatory models that externalize or individualize responsibility for inequity, thereby reflecting how attributional framing interacts with structural context.

#### 3.2.4. Communication & Behavior

Despite providers’ efforts to consciously reject stigma and prejudice, implicit bias manifests through language, empathy, and other nonverbal behaviors [30,38,47,48,49,69]. Physicians with greater implicit bias scores were more likely to use anxiety-related words or pronouns showing dominance during racially discordant interactions, contributing to a less positive patient experience [30]. Simulated encounters also demonstrated that physicians with pro-White implicit bias tended to communicate more effectively with White standardized patients than with Black patients, most often in rapport building and information gathering [47]. Studies also show that nonverbal behaviors also reflect bias. For instance, in oncology settings, implicit bias may foster patient mistrust, which in turn disrupts nonverbal synchrony and subtly shapes the dynamics of clinical encounters [48]. Collectively, these findings reveal that bias permeates micro-level communication processes, sustaining inequities in care even when overt prejudice is absent [19,49].

#### 3.2.5. Workplace Dynamics

In addition to patient-provider relationships, bias also impacts healthcare providers themselves, especially providers who belong to racial, ethnic, and immigrant backgrounds. In a survey of healthcare professionals in Germany, nurses and technologists reported the highest levels of discrimination, most commonly related to language, gender, and nationality [70,71]. In Australia, hospital employees often lacked systemic understanding of how racism contributes to challenges and barriers in the healthcare workforce, stating that racism is primarily an individual prejudice [9,72]. Workforce dynamics highlight structural inequities and how these disparities are perpetuated not only in patient care but also in provider career achievement, satisfaction, and integration. It is important to address these barriers in which institutional policies that confront systemic racism and promote equity are needed in the healthcare workforce.

### 3.3. Comparative Analysis

#### 3.3.1. Convergence: Shared Recognition of Discrimination and Gaps in Care

Across both user- and provider-focused studies, there is shared acknowledgement that discrimination and bias remain pervasive barriers to equitable healthcare. Based on the user perspective, LGBTQ+ and racial minority patients have frequent experiences of disrespect, denial of care, and misgendering with structural stigma that influences mistrust and avoidance of healthcare [4,19,25,44,51]. These same narratives are also evident on the provider side, where clinicians themselves have identified that implicit and explicit biases, whether they be based on gender, sexuality, or race, can negatively influence patient communication, decision-making, and empathy [20,23,24,57,73]. Therefore, both healthcare users and providers recognize that these inequities do not lie merely in the individual prejudice but also in systemic shortcomings. These systemic shortcomings were driven by inadequate training and institutionalized racism in healthcare structures [3,25,35,39].

Importantly, there is also convergence in the call for reform. Patients have advocated for inclusivity, identity-affirming communication, and diversity of provider representation [42,43]. Providers identify educational reform and efforts to mitigate bias as important for improving care equity [24,64]. Ultimately, the pursuit of equity necessitates a dual transformation, structural and cultural, reflecting a shared recognition that lasting change depends on reimagining both the systems and values that shape healthcare delivery.

#### 3.3.2. Divergence: Perception Gaps in Frequency and Impact of Bias

Despite the shared perspectives, there remain divergences regarding the frequency, extent, and consequences of discrimination. Studies show that patients describe bias as frequent and harmful, leading to behaviors such as avoidance of care, systemic healthcare mistrust, and negative mental health consequences [11,28,50,55]. Providers, on the other hand, underestimate the prevalence and the overall severity of these experiences and frame them as personal, single experiences versus systematic in nature [8,9,23,66]. An example of this is through a study that highlights how mental health practitioners reported positive attitudes towards LGBTQ+ patients; however, they lacked the confidence to address specific needs of these patients [31], which suggests an overall disconnect in perceived inclusivity and overall lived patient realities.

Patients highlight the intersectionality of discrimination, in that race, sexual orientation, and gender all influence healthcare experiences [2,4]. Providers tend to compartmentalize bias into categories such as only racial or only gender bias, and this limits their capacity to address the overlapping marginalizing identities of their patients [74]. The divergence underscores the empathy gap in that providers may be able to acknowledge inequity but often do not perceive its cumulative impact on patients. The differing perceptions exemplify the attributional divide described by Weiner’s Attribution Theory [36]. Patients who see discrimination as systemic, experience chronic mistrust, and providers who see bias as situational end up contributing to empathy gaps with resistance to systemic critique.

In applying Weiner’s Attribution Theory, patients frequently make external, stable attributions, often viewing discrimination as a persistent systemic problem. In contrast, providers tend to make internal, unstable attributions, attributing negative encounters to isolated misunderstandings and patient noncompliance [36]. This can contribute to the overall differing emotional responses, such as patient mistrust and provider defensiveness, which can hinder constructive dialogue and accountability.

#### 3.3.3. Structural vs. Interpersonal Bias: Systemic Mistrust vs. Individual Framing

There are differences in how healthcare users and providers perceive inequity. Patients frame discrimination as a cumulative and structural issue within healthcare systems, citing many examples of institutional racism, exclusionary policies, heteronormative assumptions, and other experiences that shape the overall level of care [2,42,50,75]. LGBTQ+ patients have reported systemic failures in both maternal and oncologic care, where they found housing assignments, policy, and language, and intake forms reinforced exclusion of their identities [9,35,42,50].

In contrast, healthcare providers tend to conceptualize bias as attitudinal and interpersonal, which focuses on individual behaviors rather than systemic inequities [62,72]. Even when acknowledging bias, clinicians often attribute disparities to patient mistrust and cultural misunderstandings rather than design flaws [19,66]. As a result, providers often fail to acknowledge or recognize their role in reinforcing and contributing to the systemic inequities, which leads them to ignore that they can be part of the solution. These findings can be interpreted through the Structural Racism Theory [2], which highlights that provider framing of inequity as interpersonal reflects a limited understanding of institutional forces that reproduce disparities. Conversely, patient framing aligns with systemic attributions consistent with minority stress and Intersectionality [16] frameworks, overall demonstrating a lived awareness of the overall structural determinants.

The literature offers various examples illustrating these intersecting perspectives. LGBTQ+ cancer patients have reported frequent misgendering, invisibility, and exclusion of their families from treatment decisions [42,43]. Oncologic providers often acknowledge the importance of empathy; however, they underestimate how implicit bias and color-blind ideologies contribute to shaping their own nonverbal communication [48,73]. Another example is in mental health care. Patients have described incidences of stigma at multiple levels of care, leading to avoidance and re-traumatization [50]. However, mental health providers have identified minimal intersectional awareness or training on LGBTQ+ health [31]. Lastly, Montalmant & Ettinger [75] found that black women’s narratives have linked maternal mortality to disparities in systemic racism, and providers often frame inequities in terms of empathy deficits and communication challenges rather than racialized structures.

### 3.4. Policy and Practice Implications

The findings of this review underscore the need for multilevel policy and practice interventions that address both interpersonal bias and structural inequities in healthcare. At the provider level, longitudinal training approaches that integrate implicit bias awareness, structural competency, and intersectionality may be more effective than one-time cultural competence workshops in improving communication and promoting identity-affirming care. Such training should emphasize reflective practice and practical skills applicable to real-world clinical encounters.

At the institutional level, healthcare organizations can advance equity by implementing inclusive policies, including standardized use of patients’ chosen names and pronouns, routine collection of sexual orientation and gender identity data, and clear nondiscrimination guidelines. Embedding equity-focused metrics into quality improvement initiatives and establishing transparent mechanisms for reporting discrimination may further strengthen accountability and patient trust.

At the system level, broader policy efforts are needed to address upstream drivers of inequity. These include enforcing nondiscrimination protections, supporting workforce diversity initiatives, and aligning accreditation and reimbursement standards with equity goals. Together, these strategies reflect the interconnected nature of bias identified in this review and emphasize that meaningful progress toward equitable healthcare requires coordinated action across multiple levels of the healthcare system.

### 3.5. Summary

Together, healthcare user and provider perspectives converge on the recognition that discrimination will undermine patient safety, outcomes, and communication. They diverge in how they perceive, attribute causes, and define discrimination. Bridging the perceptual divide between users and providers will require interventions that take into account patient voices, culturally competent medical education, and bidirectional empathy, not only transforming individual behaviors but also addressing how inequities exist systemically.

## 4. Conclusions

This comprehensive review highlights the persistent healthcare inequities observed from both the patient and provider perspectives. Findings from the first aim of this review show that LGBTQ+ and racial or ethnic minority patients with intersecting identities often experience discrimination, denial of services, misgendering, and exclusion from care decision-making processes. These experiences are not isolated incidents, but rather part of broader structural inequities [5,18,44,50,51]. Patients described bias as systemic, which is embedded in procedures, policies, and institutional norms, not just interpersonal.

The findings from this review for the second aim highlighted that, while clinicians and professionals recognize the existence of bias, its frequency, scope, and cumulative impact are often underestimated. Providers frequently frame healthcare inequities as arising from individual prejudice or patient-level factors rather than structural factors [8,9,20,23,72]. Although willingness to learn and positive attitudes of providers are reported in many studies, there are gaps in training, confidence, and empathy, especially when caring for patients with intersectional identities.

All together, these findings underscore the influence of both patients’ lived experiences of discrimination and providers’ perceptions of bias on patient trust and care quality. The synthesis of this review highlights that addressing the user and provider perspectives together is essential for improving trust, equity, and the overall quality of healthcare delivery. Therefore, multilevel strategies are needed to integrate institutional reform with provider education and patient voices.

Several limitations should be acknowledged. First, the literature included in this review is predominantly drawn from Western, high-income countries, particularly the United States, Canada, Europe, and Australia. As a result, the findings may not fully generalize to low- and middle-income countries or to healthcare systems operating under different sociopolitical, cultural, and economic conditions. Second, differences in healthcare system models may shape how bias and discrimination are experienced and addressed. For example, dynamics within privatized healthcare systems, such as those in the United States, may differ from those in publicly funded or universal healthcare systems with respect to access, continuity of care, institutional accountability, and patient-provider relationships. These structural differences may influence both the manifestation of bias and the effectiveness of proposed interventions.

Future research should build on these insights using a longitudinal, intersectional, and policy-focused approach rather than cross-sectional and attitudinal studies. Existing studies predominantly examine short-term effects of bias reduction or empathy training, leaving the long-term impact on systemic practices and provider behavior largely unexamined. Longitudinal studies should examine how bias-based intervention programs can influence provider behavior and overall patient satisfaction and health outcomes. This may clarify if reflective, empathy-based, or structural competency interventions provide sustained improvements in equitable care [76,77,78]. Additionally, research should employ intersectionality as a guiding framework to capture multiple marginalized identities. The majority of studies to date assess bias based on a single identity, or on identities as separate, such as race or gender identity [9,16,17,18]. Exploring intersecting identities, such as how Black transgender women or queer patients with disabilities navigate care systems, can reveal the behaviors and barriers to healthcare access among multiple identity axes. This approach can strengthen theoretical understanding and practical design of interventions. Lastly, there is an important need to assess the real-world impact of structural interventions, including nondiscrimination legislation, inclusive data collection, and institutional accountability mechanisms [39,68,79]. Then, evaluation of these reforms at local, state, and national levels is needed to assess the effectiveness of the intervention in reducing overall disparities and building patient trust. Studies may also assess how diverse leadership, funding initiatives, and community-based partnerships facilitate organizational change. The collective integration of theoretical frameworks allows for a multilevel understanding of inequity through the Minority Stress and Attribution theories [36], in explaining psychological mechanisms that influence patient and provider behaviors. In comparison, Structural Racism [2] and Intersectionality [16] can clarify the sociocultural and institutional environments that perpetuate these patterns. Future interventions should be theory-driven and link bias attribution retraining, stress reduction, and structural reform to generate sustainable systemic change.

By integrating patient experiences, provider perspectives, and structural factors, this review underscores that achieving healthcare equity requires coordinated, sustained action across multiple levels. Ultimately, dismantling systemic bias is not only a matter of training or policy in isolation; it demands a cultural shift, institutional accountability, and a commitment to amplifying marginalized voices. Only through such comprehensive and enduring efforts can healthcare systems ensure trust, inclusivity, and equitable outcomes for all patients.

## Figures and Tables

**Table 1:** Summary of Theoretical Frameworks.

Theory	Level of Analysis	Core Constructs	Application in Review
Minority Stress Theory [5]	Individual/ interpersonal	Chronic stigma leading to stress and health outcomes	Explain patient avoidance, coping, and mistrust
Structural Racism Theory [2]	Institutional/ systemic	Policy and organizational inequity	Frames overall provider bias as systemic, not personal
Intersectionality [16]	Cross-level	Intersecting identities and compounding marginalization	Provides clarity to the cumulative disadvantage among SGM and racial minorities
Attribution Theory [36]	Cognitive/ psychological	Internal vs. external causal reasoning	Explains the perception gaps between providers and patients

## Data Availability

No new data were created or analyzed in this study.
